# Capmatinib targeted therapy in MET-fusion driven radiation induced glioma

**DOI:** 10.3389/fonc.2026.1739413

**Published:** 2026-04-28

**Authors:** Nolan Ford, Brian J. Dlouhy, Jeremy D. Greenlee, John M. Buatti, Jason Cleppe, Deqin Ma, Kathryn L. Eschbacher, Osorio Lopes Abath Neto, Andrew Groves

**Affiliations:** 1 Carver College of Medicine, University of Iowa, Iowa City, IA, United States; 2 Department of Neurosurgery, University of Iowa, Iowa City, IA, United States; 3 Department of Radiation Oncology, University of Iowa, Iowa City, IA, United States; 4 Department of Pharmaceutical Care, University of Iowa, Iowa City, IA, United States; 5 Department of Pathology, University of Iowa, Iowa City, IA, United States; 6 Division of Hematology/Oncology, University of Iowa Stead Family Children’s Hospital, Iowa City, IA, United States

**Keywords:** capmatinib, high grade glioma (HGG), MET fusion, MET inhibitor, pediatric neuro-oncology, targeted therapy

## Abstract

**Introduction:**

Radiation-induced gliomas (RIGs) can occur in regions of the central nervous system (CNS) previously irradiated for primary malignancies including leukemia, medulloblastoma, and ependymoma. Prognosis is uniformly poor despite treatment with standard of care therapy with radiation ± alkylating chemotherapy. Recent studies have shown that a subset of patients have gene fusions in targetable receptor tyrosine kinases (RTKs) including MET, NTRK2, and RAF1. However, clinical response and outcome to targeted therapy in this patient population have not been described.

**Methods:**

We report on two patients with a history of childhood medulloblastoma who developed RIGs harboring MET fusions treated with the selective MET inhibitor capmatinib. Clinical, molecular, and radiographic data are shared to report response and overall survival. Side effects of capmatinib were evaluated and described according to Common Terminology Criteria for Adverse Events version 5 (CTCAE v5.0). A comprehensive literature review was performed to describe all published cases of MET-altered RIGs.

**Results:**

Both patients were treated with focal re-irradiation followed by off-label capmatinib targeted therapy. Drug treatment was well tolerated in both patients with the only notable side effect being peripheral edema. Magnetic resonance imaging (MRI) showed significant radiographic response [partial response in both as assessed by the Response Assessment in Pediatric Neuro-Oncology (RAPNO) criteria for high-grade glioma (≥50% decrease)]. Unfortunately, both tumors became resistant and progressed. Overall survival (OS) from diagnosis was 11 and 15 months, respectively, while median OS in historical cohorts is ~9 months. We review the characteristics of MET-altered pediatric high-grade glioma using the Open Pediatric Brain Tumor Atlas (Open PBTA) and published series, which suggests that MET fusions may be enriched in RIGs.

**Discussion:**

Our two cases highlight the promising CNS penetration and on-target activity of capmatinib in MET-altered glioma; however, the development of rapid resistance emphasizes the pressing need to develop combination and/or new therapies for RIG.

## Introduction

Central nervous system (CNS) tumors are the leading cause of cancer-related death in childhood (1). Radiation is an essential component of therapy for many tumor types; however, radiation-induced cancers are a known sequelae that can lead to significant morbidity and mortality (2, 3). Radiation-induced gliomas (RIGs) occur in regions of the CNS previously irradiated for primary malignancies including leukemia and brain tumors. Latency is variable, ranging from 2 to 40 years after initial exposure. Data from the Surveillance, Epidemiology, and End Results (SEER) Program Registry estimate that RIGs affect 1%–4% of patients who have received cranial irradiation (4). There is a linear correlation between dose of radiation and RIG development, which is especially amplified in children younger than 5 years (5). The mechanism of tumorigenesis is poorly understood, but thought to be related to DNA damage (specifically double-stranded breaks) in healthy cells exposed to radiation. RIGs follow an aggressive clinical course, with a median overall survival of ~9 months despite standard-of-care treatment with radiation ± alkylating chemotherapy (6).

Recent studies have tried to better characterize the molecular landscape of these tumors using genome, transcriptome, and methylome sequencing (7–9). These studies revealed that tumors primarily cluster with the diffuse pediatric high-grade glioma (pHGG), H3-wild type (WT) and IDH-WT, and RTK1 subtype, with recurrent alterations including PDGRFA amplification and loss of CDKN2A/B (without H3 or IDH1/2 mutations). In addition, a subset of patients were found to have gene fusions in targetable receptor tyrosine kinases (RTKs) including MET, NTRK2, and RAF1. However, clinical response and outcome to targeted therapy in this patient population have not been reported.

MET fusions have also been identified in a subset of *de novo* infant (age <3), pediatric (age 3–18), and adult high-grade gliomas (10–12). While there is no current proven targeted therapy for these patients, a recent study uncovered promising anti-tumor efficacy of the selective, CNS-penetrant MET inhibitor capmatinib in mouse models (13). Furthermore, this effect was synergistic with radiation. Herein, we report two RIG cases harboring MET fusions who were treated with radiotherapy and capmatinib. Pathologic features, imaging response, and treatment-related side effects were collected and analyzed. Finally, a comprehensive review of all published MET-altered RIGs is presented to highlight unique features compared to *de novo* pediatricpHGGs.

## Materials and methods

### Patients and samples

This study was approved by the University of Iowa Institutional Review Board (#202212193), Pediatric Oncology Specimen and Clinical Data Bank. Patients were treated at the University of Iowa Stead Family Children’s Hospital. Multisequence, multiplanar magnetic resonance imaging (MRI) was performed on a 1.5-T system with and without gadolinium (Gadavist) contrast. Histology was performed and reviewed by staff neuro-pathologists, with immunohistochemistry including IDH1-R132H, ATRX, p53, H3K27M, and H3K27me3.

### Molecular analysis

Molecular profiling of tumors was performed by the Divisions of Neuropathology and Molecular Pathology at the University of Iowa. In brief, nucleic acid was extracted from microdissected, formalin-fixed, paraffin-embedded tissue using the RNeasy FFPE minikit (Qiagen, CA). DNA sequencing was performed using a custom DNA-based cancer panel (214 genes) to detect single nucleotide variants (SNVs), small deletions/duplications, copy number variants (CNVs) and microsatellite status (MSI) as previously described (14). RNA was sequenced using the comprehensive thyroid and lung FusionPlex assay (IDT Technologies, IA). Fusions were called using Archer Analysis v6.0. Both cases were sent for methylation profiling and classification at the National Institutes of Health (NIH) using the NCI/Bethesda classifier v2.0 and Heidelberg classifier (v12b6) (15).

### Statistical analysis

Descriptive analyses and chi-square tests were performed in GraphPad Prism. Response was evaluated using the Response Assessment in Pediatric Neuro-Oncology (RAPNO) criteria for high-grade glioma (16). Overall survival was defined as the interval between diagnosis and death from any cause. Adverse events were recorded based on clinical documentation in the electronic medical record (EMR) and graded based on Common Terminology Criteria for Adverse Events version 5 (CTCAE v5.0).

## Results

### Case #1 (RIG1)

**Figure 1:**
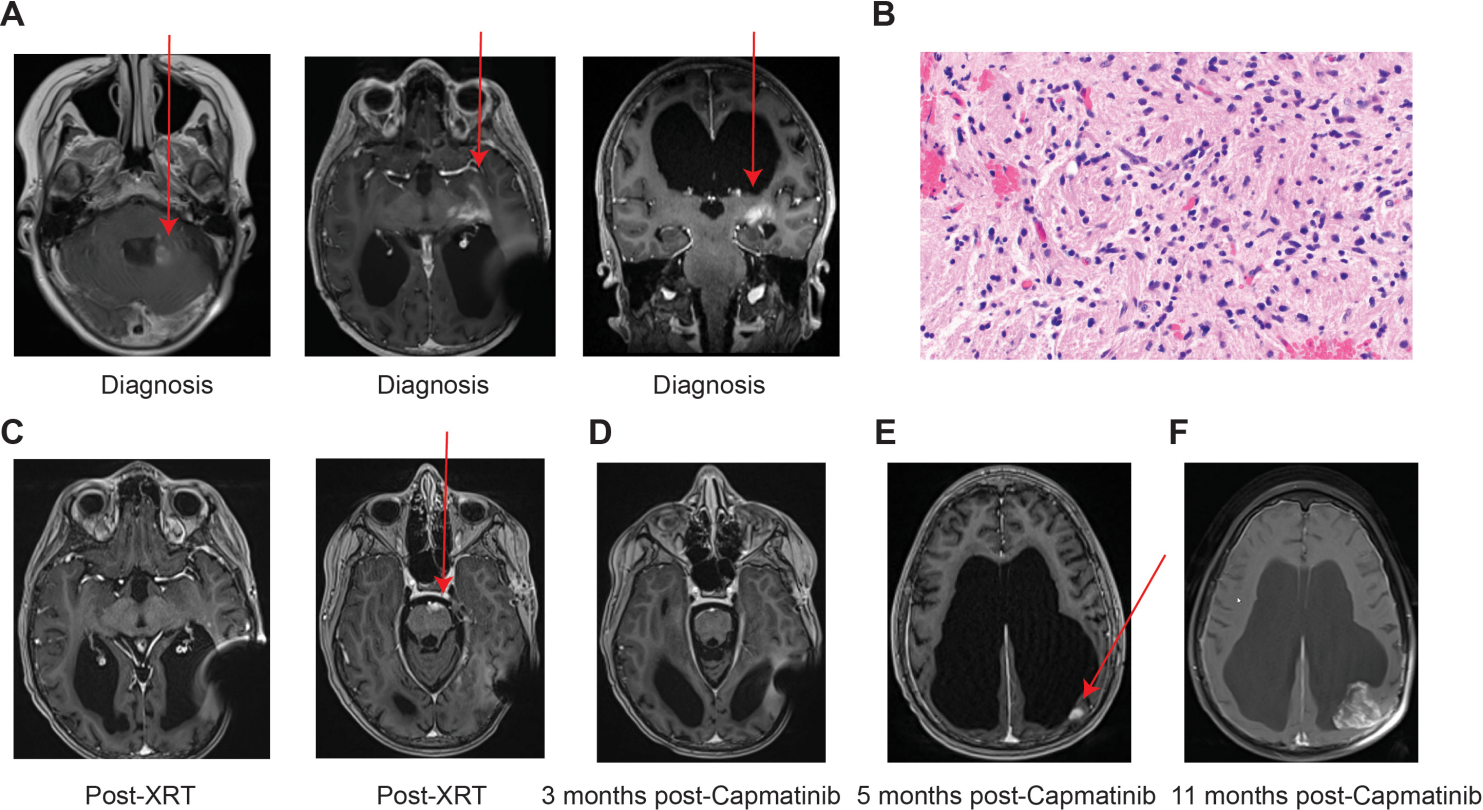
Pathology and imaging of case #1 (RIG1). **(A)** Brain MRI (T1 axial and coronal post-contrast) demonstrating enhancing left cerebellar lesion (left) and infiltrative enhancement of left temporal lobe and cerebral peduncle (middle/right) **(B)** Hematoxylin and eosin (H&E) stain, 400× magnification: Moderately cellular infiltrating glioma with irregular, elongated nuclei. **(C)** Brain MRI (T1 axial post-contrast) demonstrating decreased enhancement in left temporal lobe post-radiation (left), although new enhancing lesion in ventral pons (right). **(D)** Decreased enhancement in ventral pons 3 months after start of capmatinib. **(E)** Brain MRI (T1 axial post-contrast) demonstrating new progressive enhancing tumor in left posterior parietal lobe 5 months, then 11 months **(F)** after start of capmatinib.

RIG1 was a male patient who was initially diagnosed with M1 medulloblastoma at age 7 and received treatment with craniospinal radiation and chemotherapy as per POG9031 (vincristine, cyclophosphamide, cisplatin, and etoposide). Although not known at the time, his tumor was later sent for DNA methylation array profiling at the NIH, which matched with high confidence to medulloblastoma, non-WNT/non-SHH activated, Group 4/Subclass VII. He completed therapy with no evidence of disease and was eventually transitioned to our institutional survivorship clinic after 5 years of stable surveillance imaging. Late effects of therapy included hypothyroidism, growth hormone deficiency, hearing loss, and cataracts. At age 23, approximately 15 years s/p completion of therapy, he had a surveillance brain MRI that showed a small, sub-centimeter diffusion-restricting nodular lesion in the left cerebellar hemisphere without enhancement. A plan was made for close-interval follow-up, and unfortunately, on repeat imaging 3 months later, there was new associated contrast enhancement, with an additional area of infiltrative enhancement in the left temporal lobe extending to the left cerebral peduncle (**Figure 1A**). At the visit, the patient also reported new clinical symptoms of ataxia, with the caregiver noticing him “leaning more to the left while watching television”. Spine MRI was also obtained and did not show any other concerning sites of disease. Given the concern for recurrent medulloblastoma, additional workup with lumbar puncture was performed and did show rare, atypical cells with high nuclear:cytopasmic ratio and fine chromatin concerning for recurrent medulloblastoma. Given the low number of cells (three total nucleated cells/mm^3^), it was not feasible to create a tissue block for immunohistochemical staining or molecular testing.

As it was not possible to differentiate between recurrent medulloblastoma vs. RIG, a needle biopsy of the left temporal brain lesion was performed using Stealth navigation guidance. Pathology showed a glial neoplasm with elevated mitotic activity, and no definitive vascular proliferation or necrosis (**Figure 1B**). Next-generation sequencing (NGS) panel testing revealed a TP53 mutation and MET amplification, and RNA sequencing (RNA-seq) fusion testing detected a CAPZA2::MET fusion (**Table 1**). Methylation testing was performed at the NIH, which matched the tumor to diffuse pHGG, RTK1 subtype, with high confidence (NCI/Bethesda = 0.78, Heidelberg = 0.72). MGMT promoter methylation was not detected. Based on this diagnosis, he was treated with focal radiation to a total dose of 48 Gy in 40 fractions. Adjuvant treatment with concurrent and maintenance temozolomide was discussed and offered to the patient; however, given the toxicity profile and the patient’s prioritization of quality of life, he declined.

**Table 1:** Molecular testing for MET-altered RIG Cases #1 and #2.

Case Number	Fusion panel	Mutation panel	MGMT	DNA methylation
Case #1	CAPZA2::MET	TP53 p.C275G (VAF 47%)	No	Diffuse pediatric high-grade glioma RTK1
		MET CNG (29)		
Case #2	PTPRZ1::MET	CNG: MET (4), MDM4 (4)	No	No match, but suggestive of high-grade glioma, H3- and IDH-wild type
		CNL: AXL (1), CDKN2A (0.24),		
		CDKN2B (0.26), PPARG (1), TSC1 (1)		

CNG, copy-number gain; CNL, copy-number loss; MGMT, promoter methylation.

MRI 4 weeks after completion of radiation showed a good radiographic response in the treated regions (left cerebellar hemisphere, left temporal lobe); however, new mass-like areas of enhancement were seen in the ventral pons concerning for metastatic spread (**Figures 1A, C**). After discussion with the patient and his family, targeted therapy with capmatinib was started at the recommended adult dose of 400 mg BID with management. The patient was monitored closely for side effects, with only toxicity being Grade 2 tolerable peripheral edema (CTCAE v5.0). MRI performed 3 months after initiation of capmatinib showed complete response in the enhancing pontine lesion (**Figure 1C**). Three months later, imaging noted stable disease in known tumors; however, there was a new enhancing nodule in the left parietal lobe (**Figure 1D**). He also had worsening of his peripheral edema to intolerable grade 2 (including scrotal edema), for which his capmatinib dose was decreased to 300 mg BID. Other interventions included compression stockings and the addition of furosemide and spironolactone with subsequent mild improvement. Repeat imaging 3 months later showed significant increase in the enhancing parietal lobe nodule, and clinically, the patient developed neurologic decline including bilateral lower-extremity weakness and worsening dysmetria/ataxia (**Figure 1D**). Surgical resection was discussed with the patient and family, but given his poor functional status (Karnofsky Performance Scale = 40), they elected for palliative radiation. Unfortunately, prior to initiation of radiation, he developed acute worsening with altered mental status, profound paraplegia, and disordered breathing. Per prior do-not-resuscitate (DNR) advance directives, the patient was admitted for comfort care and passed away shortly thereafter surrounded by family and loved ones (15 months after diagnosis).

### Case #2 (RIG2)

RIG2 was a female patient who was diagnosed with M3 medulloblastoma at age 2. Treatment with chemotherapy as per COG-9921 was started; however, imaging on therapy showed progression of spinal metastases for which therapy was intensified to COG-99703 with three autologous stem cell transplants. After completing consolidation and turning 3, she was treated with craniospinal irradiation. Late effects of therapy included hypothyroidism, ovarian dysfunction, growth hormone deficiency, and hearing loss. She underwent gross-total resection of a left cerebellar tentorium meningioma approximately 21 years after completion of therapy due to slow but persistent growth over a 5-year period. Pathology was consistent with Grade 1 meningioma, for which no further treatment was recommended. On subsequent surveillance imaging (~22 years after completion of initial radiation), a new area of enhancement in the left cerebral peduncle and pons was noted.

**Figure 2:**
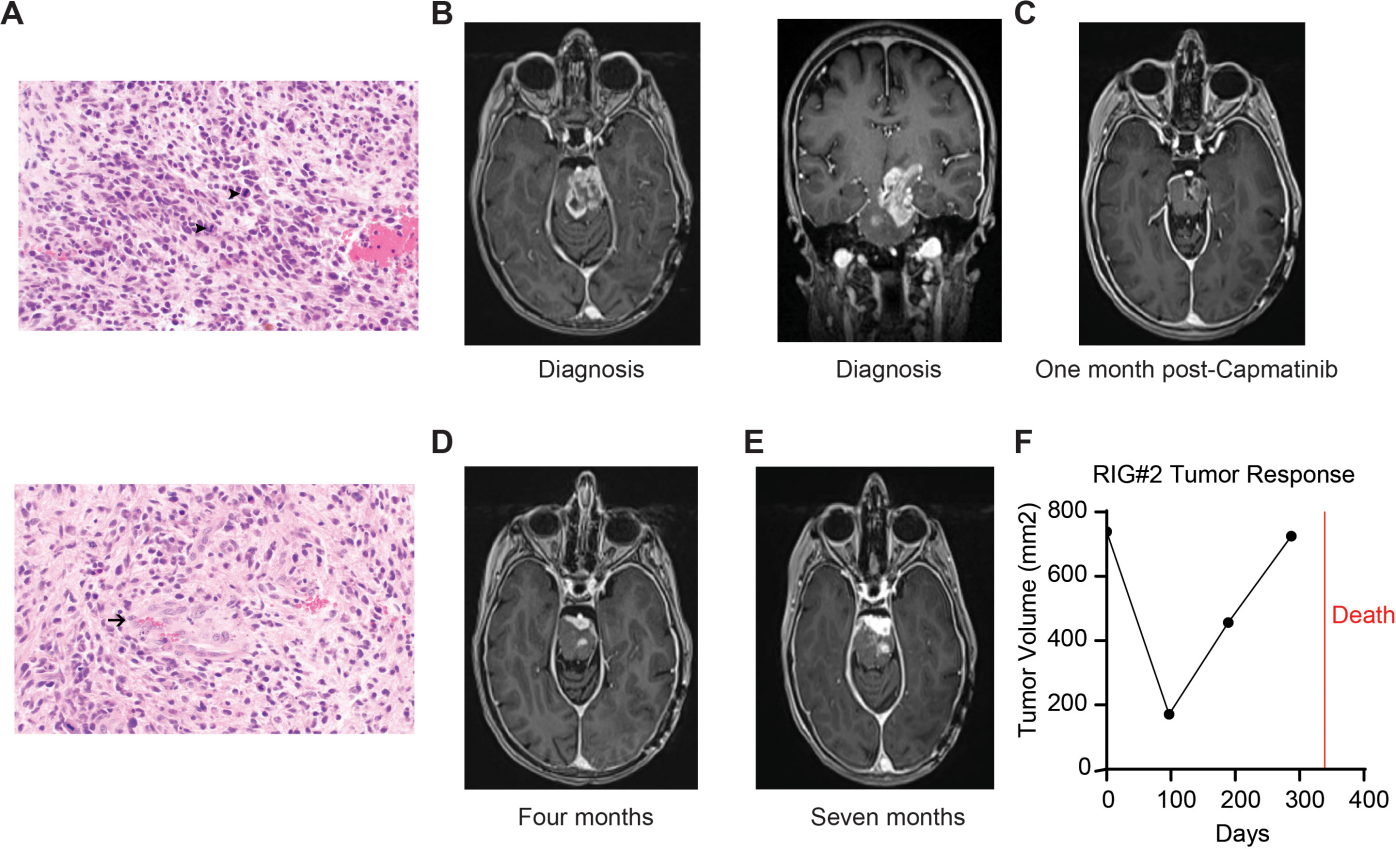
Pathology and imaging of case #2 (RIG2). **(A)** H&E stain, 400× magnification showing hypercellular infiltrating glioma with frequent mitotic figures (arrowheads, top) and microvascular proliferation (arrow, bottom). **(B)** Brain MRI (T1 axial and coronal post-contrast) demonstrating enhancing brainstem mass at diagnosis. **(C)** Brain MRI (T1 axial post-contrast) demonstrating tumor response post-radiation and 1 month after initiation of capmatinib. **(D)** Brain MRI (T1 axial post-contrast) showing progressive tumor growth at 4 and 7 **(E)** months after initiation of capmatinib. **(F)** Graph of tumor volume by MRI by day post-diagnosis.

She underwent stealth-guided needle biopsy of the mass, with pathology showing a hypercellular, infiltrating glial neoplasm with microvascular proliferation, moderate nuclear atypia, and numerous mitoses (**Figure 2A**). NGS showed numerous copy-number gains (MET and MDM4) and losses (AXL, CDKN2A, CDKN2B, PPARG, and TSC1). RNA-seq fusion testing detected a PTPRZ1::MET fusion (**Table 1**). Methylation testing was performed at the NIH, and although no consensus match was made, findings were suggestive of high-grade glioma, H3- and IDH-WT family. NCI/Bethesda classifier matched with HGAP with a confidence score of 0.71, while Heidelberg matched with the GBM_CBM class with a score of 0.69. No MGMT promoter methylation was detected. The patient elected to start treatment with focal radiation (36 Gy in 20 fractions) along with concurrent TMZ (75 mg/m^2^/day). During chemoradiotherapy, the patient developed grade 4 thrombocytopenia and grade 3 transaminitis for which temozolomide had to be held. The family decided to defer treatment with maintenance temozolomide, and instead, trial targeted therapy with capmatinib. She tolerated therapy well with the only side effect being grade 1 peripheral edema. Imaging 1 month after starting capmatinib showed dramatic tumor response (**Figure 2B**). However, the next MRI assessment 3 months later showed interval progression (**Figure 2C**) for which combination temozolomide (150 mg/m^2^ × 5 days, 28-day cycles) was added. Imaging 1 month later unfortunately showed further progression; thus, the family decides to stop therapy and enroll on hospice (**Figures 2C, D**). The patient passed away 11 months after initial diagnosis.

### Review of MET-altered RIG

There have only been a few published cases of RIGs that underwent comprehensive molecular profiling. Of these, only a small subset of cases underwent RNA-seq for fusion identification (possibly due to the logistical challenges of isolating high-quality RNA from archival tissue). Two papers, Desisto et al. and Deng et al., reported a total of 22 RIG cases that underwent RNA-seq (6, 7). Of these, six had a MET fusion for an incidence of 27% (**Table 2**). To compare this to *de novo* pHGG, we queried the Pediatric Brain Tumor Atlas (PBTA) (17). Of 151 evaluable patients with H3/IDH-WT high-grade glioma, only 3 (2%) had MET fusions. This difference in frequency was statistically significant by chi-square testing (*p* < 0.001).

**Table 2:** Review of published cases for MET-fusion-driven RIG.

Case Number	Age (first dx)	Age of RIG	Latency	Initial cancer	RIG location	TTD (months)	MET fusion partner	MGMT	Methylation	Other notable alterations
Case #1	7	23	16	MB	Temporal lobe	14	CAPZA2	No	pedRTK1	TP53mut, CDKN2A CNL
Case #2	2	25	23	MB	Brainstem	13	PTPRZ1	No	No match*	MET, MDM4 CNG, CDKN2A/B CNL
“Desisto_22”	NA	12	NA	ALL	NA	1	ITD	No	pedRTK1	CDKN2A loss
“Desisto_24”	10	23	13	MB	Brainstem + cerebellum	5	OLFM3	No	pedRTK1	CDK4 gain, TP53mut
“Desisto_27”	4	11	7	MB	Cerebellum	4	CAPZA2	No	pedRTK1	CDKN2A loss, TP53 mut, PDGGFRA CNG
“Deng_RIG09”	NA	~11–15	4	MB	Cerebellum	8	CAPZ2	Yes	pedRTK1	CDKN2A/B deletion
“Deng_RIG11”	NA	~11–15	3	MB	Cerebellum	NA	PTPRZ1	No	pedRTK1	TP53mut
“Deng_RIG25”	NA	~16–20	4	MB	Cerebellum	4	PTPRZ1	No	pedRTK1	TP53mut (germline), ATRXmut

NA, not available; MB, medulloblastoma; ALL, acute lymphoblastic leukemia; TTD, time to death; MGMT, promoter methylation; CNG, copy-number gain; CNL, copy-number loss.

*No match but suggestive of H3/IDH-wild-type high-grade glioma.

Desisto = Reference 6. Deng = Reference 7.

## Discussion

**Figure 3:**
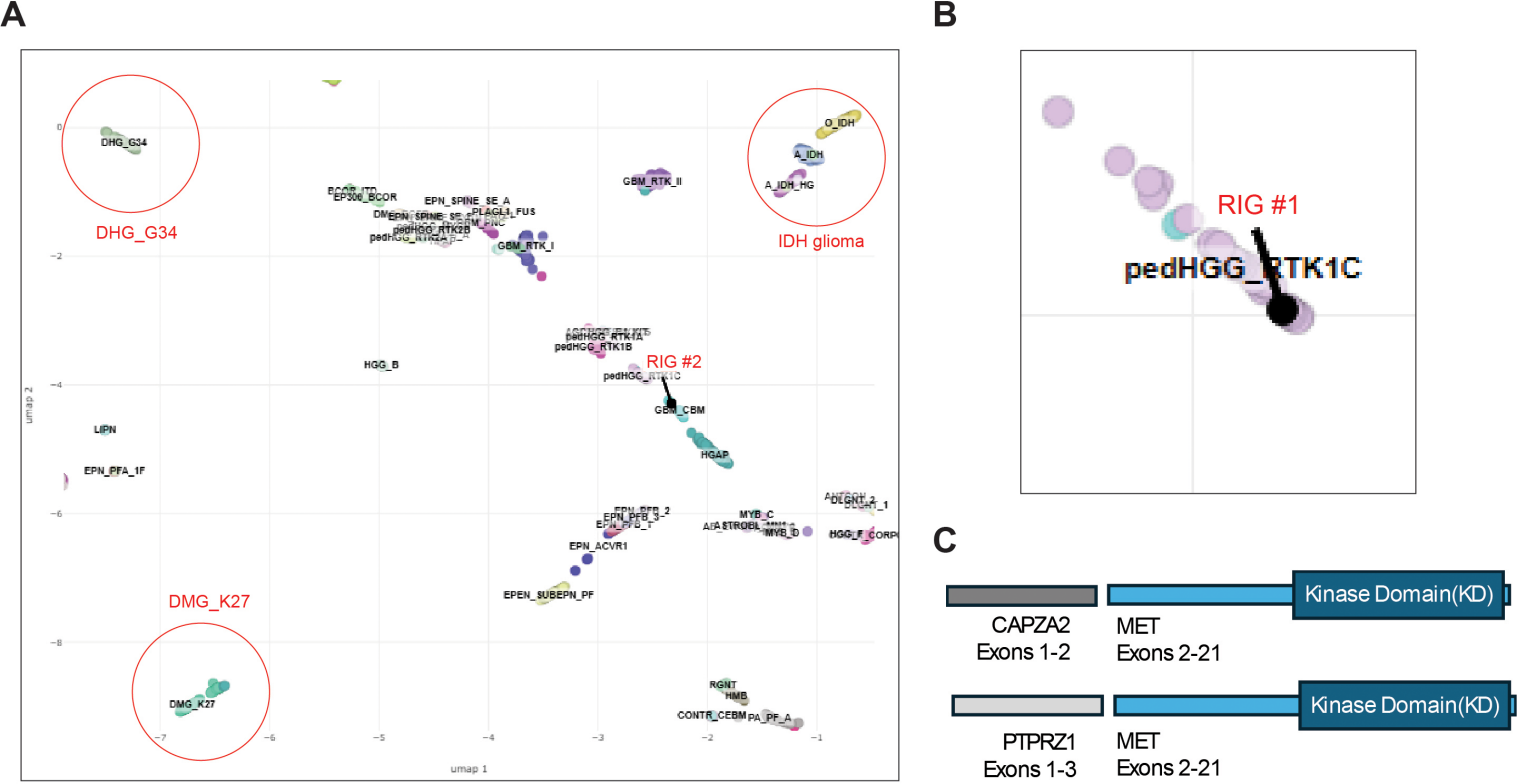
Molecular highlights of RIG cases. **(A)** UMAP projection using NCI/Bethesda classifier for case RIG#2 (highlighted in red) showing clustering within H3- and IDH-wild-type high-grade glioma. **(B)** UMAP projection using NCI/Bethesda classifier for case RIG#1 (highlighted in red) showing clustering within pedHGG_RTK1. **(C)** MET fusion breakpoints for RIG#1 (top) and RIG#2 (bottom).

RIG is a late complication of cranial radiation, which shares many features with *de novo* pHGGs; however, recent studies have shown that there are unique distinguishing molecular and clinical features (6–9, 18). RIGs have a propensity to cluster with the pHGG RTK1 subgroup, and indeed, Case #1 matched with high confidence to this subgroup while Case #2 had no specific subgroup match (although was suggestive of H3- and IDH-WT pHGG) (**Figure 3**). Focal SNV/CNVs in our cases that match with published RIG data include the following: TP53 mutation, amplification of MET, and copy-number loss of CDKN2A/CDKN2B (**Table 2**). Notable alterations seen in *de novo* pHGG that were not seen in our cases (or commonly in other RIG datasets) include H3F3A, HIST1H3B, IDH1/2, ACVR1, EGFR, or TERT promoter alterations. It is also interesting to note that neither of our cases had MGMT promoter methylation, and in the largest meta-analysis of RIG, only 28% (8/29) of tested cases had MGMT methylation (compared to ~45% in *de novo* HGG) (19). This finding may correlate with the aggressive course of RIGs.

While the number of patients/samples is small, previous studies have shown that RNAseq for fusion detection in RIGs can identify actionable targets in a high proportion of cases. As highlighted above, combing cases tested with RNA-seq by Deng et al. and Desisto et al. found a rate of MET fusion of 27% (6/22, **Table 2**), compared to 2% (3/151) in the PBTA pHGG dataset. Of note, other series (that include H3-altered HGG) have identified MET fusions in a higher percentage (~10%) of pHGG (20). Our data suggest that MET fusions may be enriched in RIGs, which is highly relevant given the availability and development of MET kinase inhibitors. The early generation multi-target tyrosine kinase inhibitors crizotinib and cabozantinib inhibit c-MET and are approved by the Food and Drug administration (FDA) for certain non-CNS cancers (21). The selective MET inhibitors capmatinib and tepotinib were more recently approved for non-small cell lung cancer (NSCLC).

In the Phase II GEOMETRY trial, capmatinib showed an impressive intracranial response rate for metastases of 54%, suggestive of promising CNS penetration (22). A recent study by Zuckermann et al. more thoroughly investigated the ability of capmatinib to cross the blood–brain barrier and exert anti-tumor effects in MET-fusion-altered pediatricHGG (13). They performed pharmacokinetic profiling of capmatinib in mice and found that it reaches 9× higher maximal concentration of unbound drug in the brain compared to crizotinib (~103 nM vs. ~11 nM). They also showed that capmatinib had impressive anti-tumor efficacy in MET-fusion-driven glioma models (*in vitro* and *in vivo*). Furthermore, there was a synergistic effect when capmatinib was combined with radiation.

Based on this promising pre-clinical data, capmatinib was selected in our two cases over earlier generation MET inhibitors crizotinib/cabozantinib due to improved selectivity. Capmatinib was chosen over tepotinib as the latter was not FDA-approved until February 2024 (after the presentation of our patients). In the two cases presented, capmatinib clearly penetrated the CNS at effective concentrations based on the rapid radiographic responses. Unfortunately, both responses were short-lived and tumor progression led to patient demise. In a study by the International Cancer Genome Consortium PedBrain Tumor Project, the authors describe a case with very similar clinical features (20). An 8-year-old boy presented 3 years post-treatment for medulloblastoma with a new cerebellar lesion that ended up being a PTPRZ1::MET fusion RIG. He was treated with crizotinib, and MRI 2 months later showed partial radiographic response (with clinical improvement); however, several new lesions were observed that rapidly progressed, leading to patient death.

Interestingly, the authors overexpressed a TFG::MET fusion in neural progenitor cells and then transfected them into the striatum of CDKN2A^−/−^, TP53^−/−^, and WT mice. The CDKN2A- and TP53-altered mice rapidly developed tumors while WT mice did not, suggesting that oncogenesis is dependent on other drivers besides MET. This matches clinical data from our cases (and other RIG datasets), showing that MET-altered RIGs frequently have other alterations (TP53 mutation, CDKN2A loss, PDGFRA and CDK4 gain, and chromothripsis). It is therefore likely that the short duration of capmatinib response was through independent oncogenic mechanisms. Another possibility is secondary MET resistance mutations, which has been described in other cancers such as NSCLC (23). For Case #2, plasma ctDNA (Tempus xF+) was tested at the time of progression to investigate possible mechanisms of resistance but did not reveal any actionable alterations (likely due to the known low level of ctDNA in plasma of patients with brain tumor due to the blood–brain barrier). Autopsy was not pursued by family in either case, which might have provided further mechanistic insights. These two cases also highlight the diagnostic dilemma facing clinicians when trying to discriminate RIGs from recurrent tumors. Two recent studies highlight the potential role that cerebrospinal fluid (CSF) ctDNA can play as a non-invasive diagnostic tool, demonstrating successful identification of RIG diagnoses (rather than recurrent medulloblastoma) using CSF liquid biopsy (24, 25). If clinically available, this technology may have avoided the need for neurosurgical biopsies in both of our RIG cases.

**Figure 4:**
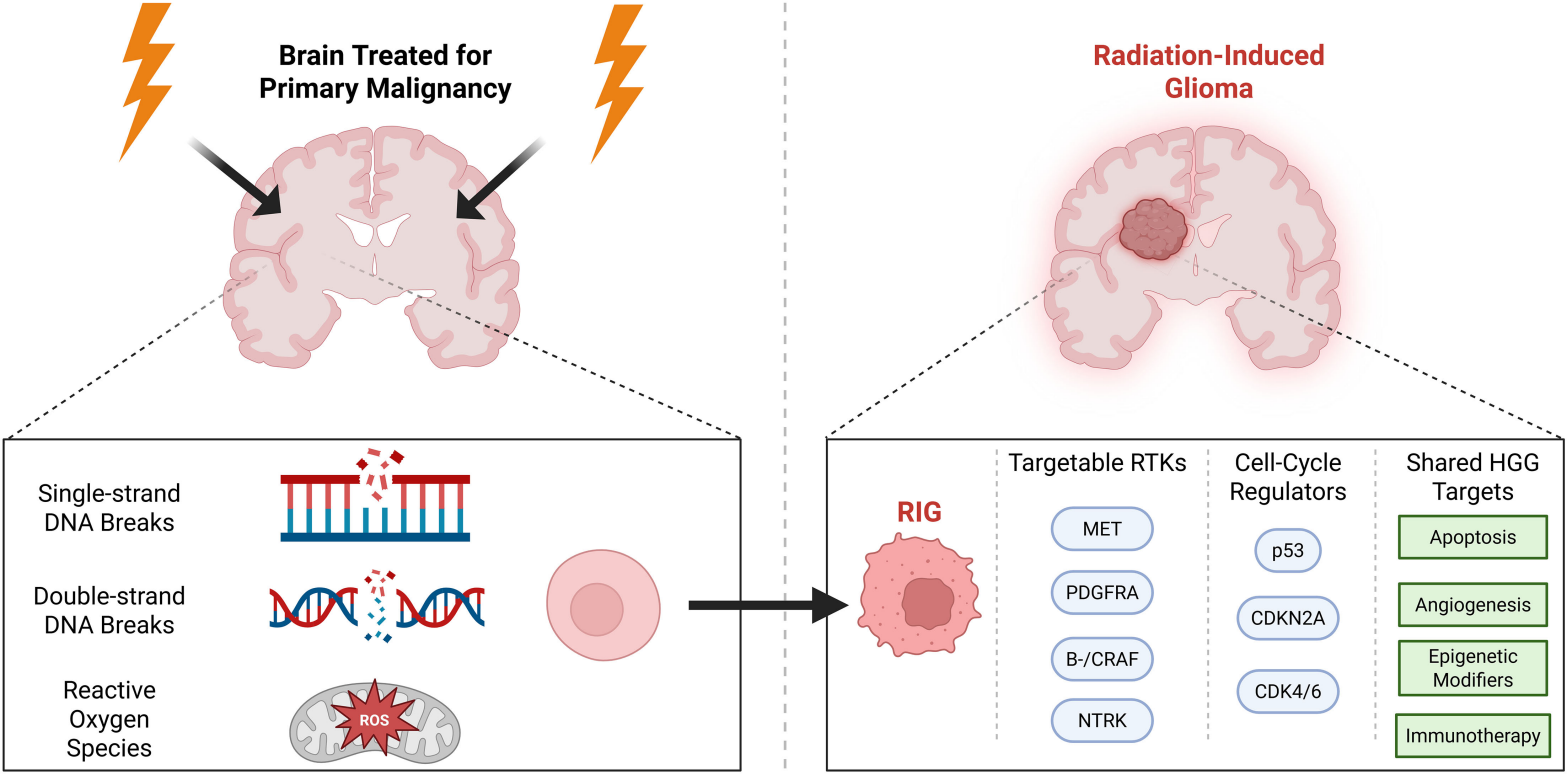
Future therapeutic directions for MET-altered RIG. Created in https://BioRender.com.

In looking to the future, it is notable that Zuckerman et al. found that capmatinib synergizes with radiation in MET-driven pHGG models, as it is possible that these two patients might have had longer responses if capmatinib was given concurrently with radiation. It is also worthwhile to review the mechanisms of MET inhibitor resistance in MET-driven NSCLC, which can include second-site MET mutations or off-target alterations (EGFR, KRAS, and HER2) (23). Strategies that have been proven effective to combat these mechanisms include “class-switching” between Type I and Type II MET inhibitors, or combining with additional inhibitors targeting secondary activated pathways (26). Although durable responses to targeted agents have been demonstrated in other pediatric brain tumors (e.g., MAPK inhibition in low-grade glioma, or ALK/NTRK/ROS inhibition in infantile high-glioma), our cases highlight that monotherapy is unlikely to be effective in MET-altered RIGs (**Figure 4**). Ultimately, our findings highlight that MET fusions are likely enriched in RIGs and capmatinib is worthy of further clinical investigation. Future studies should focus on combination strategies to combat resistance in this aggressive tumor.

## Data Availability

The original contributions presented in the study are included in the article/supplementary material. Further inquiries can be directed to the corresponding author.
